# 磨玻璃结节型肺癌胸膜改变与脏层胸膜侵犯的相关性研究进展

**DOI:** 10.3779/j.issn.1009-3419.2022.102.50

**Published:** 2022-12-20

**Authors:** 宝东 刘

**Affiliations:** 100053 北京，首都医科大学宣武医院胸外科 Department of Thoracic Surgery, Xuanwu Hospital, Capital Medical University, Beijing 100053, China

**Keywords:** 肺肿瘤, 磨玻璃结节, 胸膜改变, 脏层胸膜侵犯, Lung neoplasms, Ground-glass nodule, Pleural deformation, Visceral pleural invasion

## Abstract

脏层胸膜侵犯（visceral pleural invasion, VPI）是非小细胞肺癌（non-small cell lung cancer, NSCLC）预后的不良影响因素之一。随着计算机断层扫描（computed tomography, CT）肺癌筛查的普及，肺磨玻璃结节（ground-glass nodule, GGN）的发现越来越多，但是磨玻璃影（ground-glass opacity, GGO）型肺癌的胸膜改变与VPI之间的关系是否影响亚肺叶切除的疗效尚不明确，本文对此进行了梳理。

## Abstract (en)

Visceral pleural invasion (VPI) is one of the negative prognostic factors of non-small cell lung cancer (NSCLC). With the popularization of computed tomography (CT) screening for lung cancer, more and more ground-glass nodule (GGN) have been found. However, it remains unclear whether the relationship between the pleural deformation of lung cancer manifesting as ground-glass opacity (GGO) and VPI affects the effect of sub-lobectomy, which is reviewed in this paper.

肺癌分期的目的是为了评估治疗的预后^[1]^。国际肺癌研究协会（International Association for the Study of Lung Cancer, IASLC）制定的第8版肺癌肿瘤原发灶-淋巴结-转移（tumor-node-metastasis, TNM）分期系统中，将脏层胸膜侵犯（visceral pleural invasion, VPI）列为T分期从T1提高到T2a的重要因素之一。有研究^[2]^表明，IA1期/IA2期/IA3期的5年生存率分别为90%、85%和80%，而IB期却只有73%。VPI使肺癌TNM分期系统从IA期提高到IB期，也是IB期非小细胞肺癌（non-small cell lung cancer, NSCLC）完全切除术后需要辅助治疗的高危因素。因此，准确判断VPI对于NSCLC患者术后是否给予辅助治疗十分重要。由于VPI是磨玻璃结节（ground-glass nodule, GGN）型肺癌从T1提高到T2a的唯一因素（GGN型肺癌很少存在累及主气管、肺门、肺不张等情况，在TNM分期系统中，这几个因素权重相等），由于亚肺叶切除术中病理难以确认是否存在VPI^[3, 4]^，所以研究GGN型肺癌的胸膜改变与VPI的关系尤为重要。

## 1 胸膜解剖

胸膜是一层薄而光滑的浆膜，具有分泌和吸收等功能。其可分为互相移行的内、外两层，内层被覆于肺的表面并深入肺叶间裂内的胸膜，称为脏层胸膜或肺胸膜；外层覆盖于胸壁内面、膈上面、纵隔侧面的胸膜称作壁层胸膜。由于脏、壁两层胸膜在肺根部和上下肺韧带处互相移行，在左、右两肺表面各形成了完全闭合的潜在间隙，即胸膜腔。

脏层胸膜的组织学结构包括五层：从外到内依次为间皮层、结缔组织、弹力纤维层（内、外弹力层及两者间数量不等的血管、淋巴管和纤维结缔组织）、结缔组织层（它将弹力层与肺实质分隔）。壁层胸膜的组织学结构也包括五层：从内到外依次为间皮层和疏松的结缔组织、弹力纤维层和疏松的结缔组织、胸内筋膜、骨骼肌纤维。

## 2 肺癌胸膜侵犯

早在20世纪中叶，VPI就被认为是肺癌的不良预后因素^[5, 6]^，原因可能是VPI作为肿瘤细胞的转移通路，侵犯胸膜下淋巴管，进入肺门淋巴结，再转移到纵隔淋巴结。在一篇*meta*分析^[5]^中，共纳入13项研究中的27, 171例患者（伴VPI 5, 821例，占比21%），利用优势比对不同肿瘤大小（肿瘤直径0-3 cm、3 cm-5 cm、5 cm-7 cm）伴或者不伴VPI的NSCLC患者，比较其5年总生存期（overall survival, OS），结果证实VPI是导致NSCLC预后不良的重要因素之一。与直径3 cm-5 cm（不管是否伴有VPI）的肿瘤相比，直径≤3 cm伴VPI患者生存率更高（OR=1.31, 95%CI: 1.19-1.45, *P* < 0.001）。进一步研究发现，与肿瘤直径3 cm-5 cm不伴VPI的肿瘤患者相比，伴VPI者无生存获益（OR=1.16, 95%CI: 0.95-1.43, *P*=0.15）；但是肿瘤直径3 cm-5 cm伴VPI的肿瘤患者预后不如≤3 cm伴VPI者。作者的结论是在淋巴结转移阴性的NSCLC中，VPI和肿瘤大小的协同作用影响患者的生存期，IB期NSCLC和较大肿瘤伴VPI的患者需要考虑术后辅助化疗。

## 3 肺癌胸膜侵犯的组织学分期与预后

VPI作为一个独立概念出现在国际抗癌联盟（International Union Against Cancer, UICC）TNM分期系统中还是20世纪70年代中期。1988年Hammar第一次提出了肺癌VPI的分级方法。随后，日本肺癌学会（Japan Lung Cancer Society）大体认同Hammar分级方法，并将VPI定义为肿瘤侵犯突破弹力层。2009年的IASLC肺癌第7版TNM分期系统中提出了“改良Hammar分级方法”，将VPI定义为肿瘤侵犯超过了脏层胸膜弹力层（PL1）或者侵犯了脏层胸膜表面（PL2）^[7]^。肺癌第8版TNM分期系统沿用了第7版分期系统中的内容。

PL0：镜下肿瘤细胞侵犯但未突破脏层胸膜弹力层；PL1：镜下肿瘤细胞侵犯并突破脏层胸膜弹力层，但未侵犯间皮层，即脏层胸膜表面（推荐当HE染色不能明确PL0或PL1时，建议使用弹力纤维染色诊断）；PL2：镜下肿瘤细胞侵犯间皮层，即脏层胸膜表面；PL3：镜下肿瘤细胞侵犯壁层胸膜。鉴于壁层胸膜解剖变异性大，将PL3进一步细分难以实现。

IASLC第8版TNM分期系统中，T分期修订草案明确：PL0为T1期，PL1和PL2为T2期，PL3为T3期。如果伴VPI且肿瘤直径≤5 cm，则被归类为T2a；如果伴VPI且肿瘤直径 > 5 cm但 < 7 cm，被归类为T2b；如果伴VPI且肿瘤直径 > 7 cm，被归类为T3^[7]^。现有的文献表明，即使校正了肿瘤大小，伴有VPI仍然是预后更差的重要因素，因此没有必要修改现存的VPI分级方法，区分PL1和PL2是合理的，将伴有VPI提高到T2期是合适的。同时草案也提出，将VPI进一步细化分析发现，肿瘤直径3 cm-4 cm伴VPI的肿瘤预后与4 cm-5 cm者相似，4 cm-5cm伴VPI的肿瘤预后与5 cm-7 cm者相似，因此可以将伴有VPI肿瘤的分期提高到下一个T分期，然而此方法在临床分期中的可重复性尚待明确。Yoshida等^[8]^回顾性分析了手术切除的9, 758例NSCLC资料，根据VPI的定义分为五组： < 2 cm无VPI； < 2 cm伴VPI和2.1 cm-3 cm无VPI；2.1 cm-3 cm伴VPI和3.1 cm-5 cm无VPI；3.1 cm-5 cm伴VPI和5.1 cm-7 cm无VPI；5.1 cm-7 cm伴VPI和 > 7 cm无VPI或T3肿瘤。该作者建议对7 cm以下肿瘤，伴VPI应在未来版本的TNM分期系统中升级到下一个T分期。

在一项*meta*分析^[9]^中，入选16篇文献，共16, 916例患者，其中PL1有3, 667例，PL2有1, 512例。与PL0相比，PL1或PL2的总生存率降低（HR=1.555, 95%CI: 1.399-1.730; HR=2.447, 95%CI: 1.913-3.130），且PL2明显低于PL1（HR=1.287, 95%CI: 1.114-1.487）。PL1或PL2的5年生存率低于PL0（OR=0.515, 95%CI: 0.415-0.640; OR=0.441, 95%CI: 0.336-0.579），PL2明显低于PL1（OR=0.706, 95%CI: 0.545-0.915）。因此VPI的程度影响行手术切除的NSCLC患者的预后。

2004年，Shimizu等^[10]^和Osaki等^[11]^的研究得出同样的结论：PL1和PL2的生存率没有差异。Shimizu等^[12]^在VPI定义的基础上分析超过1, 600例患者，建议对伴有VPI的直径≤3 cm的肿瘤归属于T2肿瘤，但肿瘤直径 > 3 cm应该升级到T3。然而国内的研究^[13, 14]^发现，不论在总体人群还是淋巴结阴性人群，PL0和PL1群体的预后相当，而PL2群体的预后[无进展生存期（progression-free survival, PFS）还是OS]更差。

从以上研究可以看出，VPI的程度是影响NSCLC预后的重要因素，同时PL0和PL1间生存率差异不显著。

## 4 GGN型肺癌的胸膜改变

### 4.1 GGN的恶性概率

GGN又称为亚实性结节（sub-solid nodule, SSN），是指所有含磨玻璃影（ground-glass opacity, GGO）的肺结节，又分为纯GGN（pure GGN, pGGN）和混合性GGN（mixed GGN, mGGN），也称为部分实性结节（part-solid nodule, PSN）。

#### 4.1.1 大小

八项大宗筛查试验^[15]^发现，≤5 mm恶性概率 < 1%；5 mm-10 mm恶性概率为6%-28%； > 20 mm恶性概率为64%-82%。pGGN恶性概率（59%-73%）远高于实性结节（7%-9%）。更新数据^[16]^发现，在计算机断层扫描（computed tomography, CT）筛查人群中发现肺结节大小与恶性概率显著相关：实性结节中， < 5 mm恶性概率低于1%，5 mm-9 mm恶性概率为2.2%-6%，而日本的研究提示亚厘米病灶恶性概率高于20%，远高于美国。

在NELSON研究中^[17]^，肺结节体积≥300 mm^3^，恶性概率平均为16.9%，结节直径≥10 mm，恶性概率平均为15.2%；肺结节体积100 mm^3^-300 mm^3^，恶性概率平均为2.4%，结节直径5 mm-10 mm，恶性概率平均为1.3%；肺结节体积 < 100 mm^3^，恶性概率平均为0.6%，结节直径5 mm，恶性概率平均为0.4%。

#### 4.1.2 密度

Henschke等^[18]^在基线筛查的233例阳性结果中发现，44例（19%）为SSN（16例PSN，28例pGGN），恶性概率为34%（15例），实性结节仅7%，PSN为63%（10/16），pGGN为18%（5/28）， > 20 mm结节的恶性概率为80%。

#### 4.1.3 形态

有分叶、毛刺、血管集束征（扭曲/扩张）、胸膜改变（胸膜牵拉、胸膜尾征、胸膜附着、胸膜凹陷）等征象恶性概率高^[16-23]^。

### 4.2 GGN型肺癌的胸膜改变

针对GGN来说，T2仅涉及VPI，VPI在GGN的CT胸膜改变中扮演着完全不同的角色，术前很难通过CT或磁共振成像（magnetic resonance imaging, MRI）获得VPI的准确评估，术中的肉眼判断也是不可靠的。但是，有研究^[24-26]^发现pGGN没有发生VPI，而PSN的CT表现为胸膜接触、胸膜增厚、实性成分比（consolidation tumor ratio, CTR） > 50%、结节直径 > 20 mm是VPI的主要预测指标。

#### 4.2.1 伴VPI GGN型肺癌的预后

有研究^[27-29]^对466例手术切除的直径 < 30 mm的NSCLC（237例PSN，209例pGGN）评估预后因素，发现VPI不是PSN的主要预后因素，建议对GGN型肺癌伴VPI不应升级TNM分期。Fu等^[30]^的*Cox*比例风险模型显示，I期NSCLC的无复发生存相关的独立预后因素包括年龄（HR=1.012, *P*=0.036）、磨玻璃样成分（HR=0.426, *P* < 0.001）、肿瘤大小（HR=1.348, *P* < 0.001）、病理类型（*P*=0.024）、淋巴脉管浸润（HR=1.638, *P*=0.011）、VPI（HR=1.366, *P*=0.022）。进一步分析发现，VPI在GGN组中并不是预后因子（HR=1.234, 95%CI: 0.656-2.320, *P*=0.514），并且VPI与肿瘤大小（< 1 cm、1 cm-2 cm、2 cm-3 cm、3 cm-4 cm）无关。

Zhao等^[31]^的研究共纳入156例患者，其中38例经病理证实为VPI，其将胸膜改变分为三种类型：胸膜凹陷、胸膜附着、胸膜靠近；VPI的发生率分别为38.1%、25.5%和5.3%（*P*=0.001），并且所有病例均为PL0和PL1，未见PL2病例。作者认为VPI在GGN中常见，但影像学表现不能准确地预测是否存在VPI，并且PL2发生率较低。

#### 4.2.2 伴VPI GGN型肺癌的胸膜改变发生机制

胸膜改变的生理因素可能与结节影响肺跨膜压有关；从病理学角度看，与结节周围纤维组织反应性增生使胸膜收缩，脏壁层胸膜之间形成含液体和临近肺组织的空腔有关^[32]^。

#### 4.2.3 伴VPI GGN型肺癌的胸膜改变分型

多数学者根据GGN型肺癌胸膜改变的影像学表现分为三型^[32-35]^：1型是肺窗上表现为一个或多个线性标识；2型是肺窗及纵隔窗上表现为一个或多个线性标识，胸膜侧有软组织成分；3型是肺窗及纵隔窗上表现为一个或多个软组织索条标识。此种分型方法衍生出其他一些分类方法^[31, 36, 37]^，各分型之间VPI的发生率并不一致。为此作者根据上述分型方法和临床经验，建议首先将结节是否附着脏层胸膜分为两型（均是薄层CT扫描肺窗所见）：未附着和附着；进一步将前者分为：胸膜牵拉（表现为结节与胸膜之间一个或多个线性联结）、胸膜尾征（表现为结节与胸膜之间一个或多个线性联结，胸膜侧有软组织成分）；后者分为胸膜附着（表现为结节直接附着于胸膜）、胸膜凹陷（表现为结节直接附着于胸膜，并且存在脏层胸膜凹陷）（图 1）。

**图 1:**
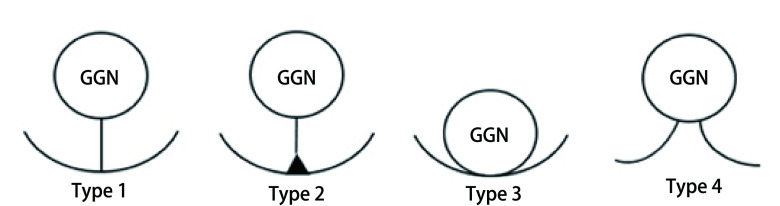
磨玻璃结节型肺癌胸膜改变示意图 Schema of pleural deformation in lung cancer manifesting as ground-glass opacity. Type 1: pleural retraction; Type 2: pleural tag; Type 3: pleural attachment; Type 4: pleural indentation. GGN: ground-glass nodule.

## 5 伴胸膜改变GGN型肺癌的手术方式

由于伴胸膜改变GGN型肺癌很少出现PL2，而PL1与PL0的预后没有不显著。同时，日本开展的前瞻性多中心观察性研究证实了GGN的影像学可以预测病理非浸润性，因此结节大小和CTR是影响预后的主要因素；肿瘤直径≤2 cm、CTR≤0.25的结节无淋巴结转移、无脉管浸润的特异性可以达98.7%，5年生存率为97.1%^[38]^；肿瘤直径≤3 cm、CTR≤0.5为局限性切除的候选者，术后5年生存率约为97%^[39]^。基于这些研究，日本开展了一系列影像学指导下的关于手术方式的前瞻性研究：JCOG0804/WJOG4507L研究，对位于肺外1/3、直径≤2 cm、CTR≤0.25的GGN可以采用楔形切除，5年无复发生存率达99.7%^[40]^；JCOG0802/WJOG4607L研究的标准是：位于肺外1/3、直径≤2 cm、CTR > 0.5的GGN行肺段切除或肺叶切除加肺门和纵隔淋巴结清扫，如果术中冰冻病理诊断或细胞学检查确认切缘有肿瘤细胞、前哨淋巴结转移，或者特殊的病理亚型[包括微乳头和实性成分，以及气腔播散（spread through air space, STAS）]等高危因素时，应进行扩大切除。结果显示：肺段切除组5年总体生存率高于肺叶切除组（94.3% *vs* 91.1%，非劣性单侧*P* < 0.000, 1，优效性*P*=0.008, 2），两组5年无复发生存率几乎完全一致（88.0% *vs* 87.9%, *P*=0.988, 9），肺段切除组的局部复发率明显高于肺叶切除组（10.5% *vs* 5.4%, *P*=0.001, 8）^[4]^；JCOG1211研究：对包括肺内2/3、直径≤2 cm、CTR > 0.25且≤0.5或直径2 cm-3 cm、CTR≤0.5的GGN行肺段切除加肺门和肺内淋巴结清扫，而不强制纵隔淋巴结清扫。结果显示：在肿瘤直径介于2 cm-3 cm而CTR≤0.5的人群中，肺段切除的5年无复发生存率达到98%；而另一人群的研究结果其实已经包含在JCOG0802/WJOG4607L研究中，因为该研究的前4年入组的肿瘤标准为直径≤2 cm、CTR > 0.25且≤0.5^[4]^。以上研究提示，大小和密度以及特殊病理学类型是影响GGN型肺癌的主要因素，而VPI对预后几乎没有影响。

2022年Altorki教授在世界肺癌大会上公布了CALGB140503研究的主要终点。研究显示，亚肺叶切除组（58.8%为楔形切除）和肺叶切除组的5年无复发生存率分别为63.6%和64.1%（HR=1.01, 90%CI: 0.83-1.24, *P*=0.017, 6）；两组5年总生存率均约为80%。CALGB140503研究中纳入肺腺癌占比63.7%，鳞癌占比为14.1%，其他预后很差的NSCLC类型占比超过20%。可以推测得出，CALGB140503研究纳入的肺腺癌可能大多数不含有磨玻璃成分，即便如此，亚肺叶切除组手术效果也不劣于肺叶切除组。所以直径≤2 cm的肺结节，不管是否伴有VPI，均不影响预后，即便是亚肺叶切除，也可以获得良好生存。

总之，虽然VPI对NSCLC的预后有不良影响，但是初步结果显示，伴胸膜改变GGN型肺癌很少出现PL2，而PL1与PL0的预后差异不显著（可以将GGN型肺癌列为T1分期），所以VPI对GGN型肺癌进行亚肺叶切除的预后影响比较小，但是仍需要进行大样本多中心临床研究。

## References

[1] Liu BD, Zhi XY (2016). Interpretation and prospect of the eighth edition of TNM staging for lung cancer. Shou Du Yi Ke Da Xue Xue Bao.

[2] Goldstraw P, Chansky K, Crowley J (2016). The IASLC lung cancer staging project: proposals for revision of the TNM stage groupings in the forthcoming (Eighth) edition of the TNM classification for lung cancer. J Thorac Oncol.

[3] Liu BD (2019). Diagnosis and treatment of pulmonary ground-glass nodules. Zhongguo Fei Ai Za Zhi.

[4] Saji H, Okada M, Tsuboi M (2022). Segmentectomy versus lobectomy in small-sized peripheral non-small-cell lung cancer (JCOG0802/WJOG4607L): a multicentre, open-label, phase 3, randomised, controlled, non-inferiority trial. Lancet.

[5] Jiang L, Liang W, Shen J (2015). The impact of visceral pleural invasion in node-negative non-small cell lung cancer: a systematic review and *meta*-analysis. Chest.

[6] Huang H, Wang T, Hu B (2015). Visceral pleural invasion remains a size-independent prognostic factor in stage I non-small cell lung cancer. Ann Thorac Surg.

[7] Travis WD, Brambilla E, Rami-Porta R (2008). Visceral pleural invasion: pathologic criteria and use of elastic stains: proposal for the 7^th^ edition of the TNM classification for lung cancer. J Thorac Oncol.

[8] Yoshida J, Nagai K, Asamura H (2009). Visceral pleura invasion impact on non-small cell lung cancer patient survival: its implications for the forthcoming TNM staging based on a large-scale nation-wide database. J Thorac Oncol.

[9] Wang T, Zhou C, Zhou Q (2017). Extent of visceral pleural invasion affects prognosis of resected non-small cell lung cancer: A *meta*-analysis. Sci Rep.

[10] Shimizu K, Yoshida J, Nagai K (2004). Visceral pleural invasion classification in non-small cell lung cancer: a proposal on the basis of outcome assessment. J Thorac Cardiovasc Surg.

[11] Osaki T, Nagashima A, Yoshimatsu T (2004). Visceral pleural involvement in nonsmall cell lung cancer: prognostic significance. Ann Thorac Surg.

[12] Shimizu K, Yoshida J, Nagai K (2005). Visceral pleural invasion is an invasive and aggressive indicator of non-small cell lung cancer. J Thorac Cardiovasc Surg.

[13] Liang RB, Li P, Li BT (2021). Modification of pathologic T classification for non-small cell lung cancer with visceral pleural invasion: data from 1, 055 cases of cancers ≤3 cm. Chest.

[14] Chen TY, Jin RR, Hua HJ (2016). Pleural invasion in lung cancer: diagnosis and research progress. Zhonghua Bing Li Xue Za Zhi.

[15] Wahidi MM, Govert JA, Goudar RK (2007). Evidence for the treatment of patients with pulmonary nodules: when is it lung cancer?: ACCP evidence-based clinical practice guidelines (2^nd^ edition). Chest.

[16] Gould MK, Donington J, Lynch WR (2013). Evaluation of individuals with pulmonary nodules: when is it lung cancer? Diagnosis and management of lung cancer, 3^rd^ ed: American College of Chest Physicians evidence-based clinical practice guidelines. Chest.

[17] Horeweg N, van der Aalst CM, Vliegenthart R (2013). Volumetric computed tomography screening for lung cancer: three rounds of the NELSON trial. Eur Respir J.

[18] Henschke CI, Yankelevitz DF, Mirtcheva R (2002). CT screening for lung cancer: frequency and significance of part-solid and nonsolid nodules. AJR Am J Roentgenol.

[19] Mets OM, de Jong PA, Scholten ET (2017). Subsolid pulmonary nodule morphology and associated patient characteristics in a routine clinical population. Eur Radiol.

[20] Xiang W, Xing Y, Jiang S (2014). Morphological factors differentiating between early lung adenocarcinomas appearing as pure ground-glass nodules measuring ≤10 mm on thin-section computed tomography. Cancer Imaging.

[21] Liang J, Xu XQ, Xu H (2015). Using the CT features to differentiate invasive pulmonary adenocarcinoma from pre-invasive lesion appearing as pure or mixed ground-glass nodules. Br J Radiol.

[22] Gao F, Sun Y, Zhang G (2019). CT characterization of different pathological types of subcentimeter pulmonary ground-glass nodular lesions. Br J Radiol.

[23] Wu F, Tian SP, Jin X (2017). CT and histopathologic characteristics of lung adenocarcinoma with pure ground-glass nodules 10 mm or less in diameter. Eur Radiol.

[24] Ahn SY, Park CM, Jeon YK (2017). Predictive CT features of visceral pleural invasion by T1-sized peripheral pulmonary adenocarcinomas manifesting as subsolid nodules. AJR Am J Roentgenol.

[25] Zhao Q, Wang JW, Yang L (2019). CT diagnosis of pleural and stromal invasion in malignant subpleural pure ground-glass nodules: an exploratory study. Eur Radiol.

[26] Ding HD, Shi JY, Zhou X (2016). Correlation analysis of preoperative imaging characteristics with visceral pleural invasion in lung adenocarcinoma presented with ground glass opacity. Wai Ke Yan Jiu Yu Xin Ji Shu.

[27] Hattori A, Suzuki K, Matsunaga T (2014). Visceral pleural invasion is not a significant prognostic factor in patients with a part-solid lung cancer. Ann Thorac Surg.

[28] Heidinger BH, Schwarz-Nemec U, Anderson KR (2019). Visceral pleural invasion in pulmonary adenocarcinoma: Differences in CT patterns between solid and subsolid cancers. Radiol Cardiothorac Imaging.

[29] Okada S, Hattori A, Matsunaga T (2021). Prognostic value of visceral pleural invasion in pure-solid and part-solid lung cancer patients. Gen Thorac Cardiovasc Surg.

[30] Fu F, Zhang Y, Wen Z (2019). Distinct prognostic factors in patients with stage I non-small cell lung cancer with radiologic part-solid or solid lesions. J Thorac Oncol.

[31] Zhao LL, Xie HK, Zhang LP (2016). Visceral pleural invasion in lung adenocarcinoma ≤3 cm with ground-glass opacity: a clinical, pathological and radiological study. J Thorac Dis.

[32] Onoda H, Higashi M, Murakami T (2021). Correlation between pleural tags on CT and visceral pleural invasion of peripheral lung cancer that does not appear touching the pleural surface. Eur Radiol.

[33] Li M, Ito H, Wada H (2004). Pit-fall sign on computed tomography predicts pleural involvement and poor prognosis in non-small cell lung cancer. Lung Cancer.

[34] Hsu JS, Han IT, Tsai TH (2016). Pleural tags on CT scans to predict visceral pleural invasion of non-small cell lung cancer that does not abut the pleura. Radiology.

[35] Meng Y, Gao J, Wu C (2022). The prognosis of different types of pleural tags based on radiologic-pathologic comparison. BMC Cancer.

[36] Kim HJ, Cho JY, Lee YJ (2019). Clinical significance of pleural attachment and indentation of subsolid nodule lung cancer. Cancer Res Treat.

[37] Fu CH, Jiang YH, Ge JY (2022). Clinical characteristics and risk factors analysis for visceral pleural invasion in mixed ground-glass nodular lung adenocarcinoma. Zhongguo Fei Ai Za Zhi.

[38] Suzuki K, Koike T, Asakawa T (2011). A prospective radiological study of thin-section computed tomography to predict pathological noninvasiveness in peripheral clinical IA lung cancer (Japan Clinical Oncology Group 0201). J Thorac Oncol.

[39] Asamura H, Hishida T, Suzuki K (2013). Radiographically determined noninvasive adenocarcinoma of the lung: survival outcomes of Japan Clinical Oncology Group 0201. J Thorac Cardiovasc Surg.

[40] Suzuki K, Watanabe SI, Wakabayashi M (2022). A single-arm study of sublobar resection for ground-glass opacity dominant peripheral lung cancer. J Thorac Cardiovasc Surg.

